# Supplementary material for: Vasoactive pharmacological management according to SCAI class in patients with acute myocardial infarction and cardiogenic shock
Source: PLoS One. 2022 Aug 4;17(8):e0272279. doi: 10.1371/journal.pone.0272279 (PMC9352108; doi:10.1371/journal.pone.0272279)
Supplement: S1 Fig — Hemodynamic in terms of A) mean arterial blood pressure B) Venous oxygen saturation during first 48 hours of CICU admission for each SCAI class. (DOCX) [file pone.0272279.s001.docx]

**S1. Hemodynamic in terms of A) mean arterial blood pressure B) Venous oxygen saturation during first 48 hours of CICU admission for each SCAI class**

**
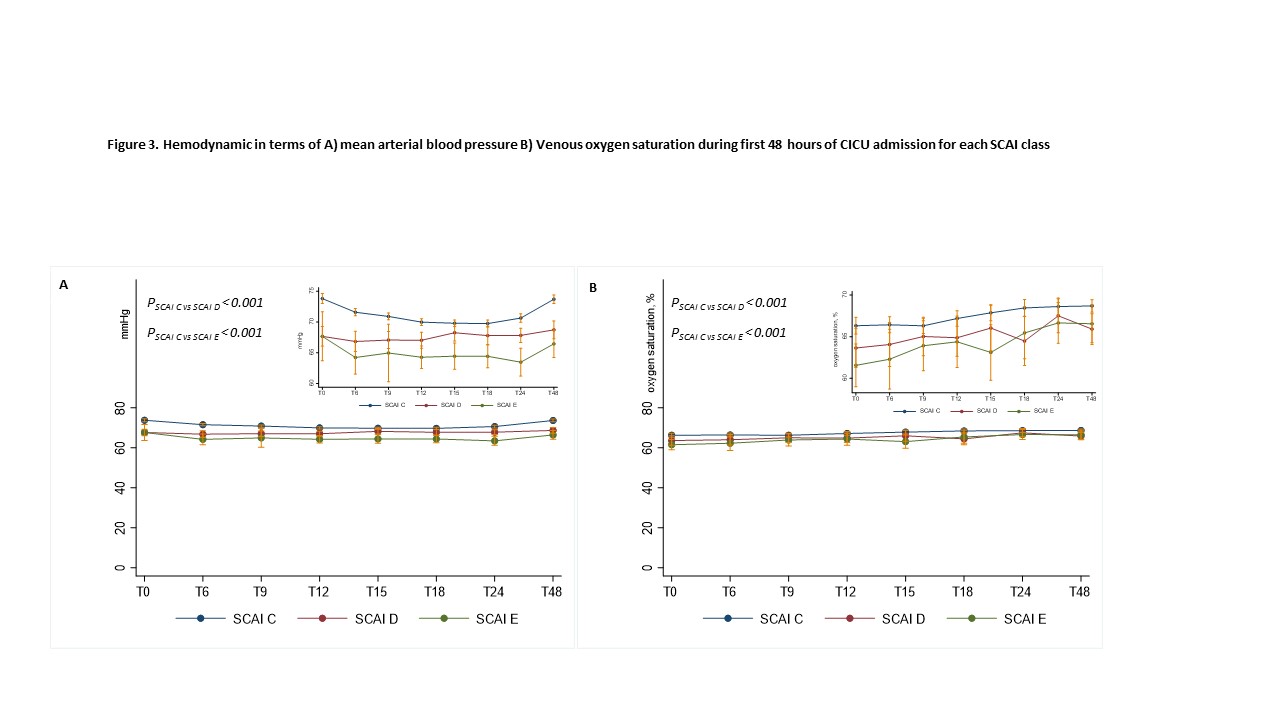
**
