# Supplementary material for: Vasoactive pharmacological management according to SCAI class in patients with acute myocardial infarction and cardiogenic shock
Source: PLoS One. 2022 Aug 4;17(8):e0272279. doi: 10.1371/journal.pone.0272279 (PMC9352108; doi:10.1371/journal.pone.0272279)
Supplement: S2 Fig — NE: norepinephrine, DA: dopamine, MIX+AD: Epinephrine and norepinephrine and/or dopamine. (DOCX) [file pone.0272279.s002.docx]

**S2. Mean VIS and mean arterial blood pressure over time according to inopressor choice in each SCAI class. NE: norepinephrine, DA: dopamine, MIX+AD: Epinephrine and norepinephrine and/or dopamine**
