# Supplementary material for: Vasoactive pharmacological management according to SCAI class in patients with acute myocardial infarction and cardiogenic shock
Source: PLoS One. 2022 Aug 4;17(8):e0272279. doi: 10.1371/journal.pone.0272279 (PMC9352108; doi:10.1371/journal.pone.0272279)
Supplement: S3 Fig — (DOCX) [file pone.0272279.s003.docx]

**S3. Mean heart rate and mean arterial lactate according to inopressor choice in each SCAI class**
