# Supplementary material for: Vasoactive pharmacological management according to SCAI class in patients with acute myocardial infarction and cardiogenic shock
Source: PLoS One. 2022 Aug 4;17(8):e0272279. doi: 10.1371/journal.pone.0272279 (PMC9352108; doi:10.1371/journal.pone.0272279)
Supplement: S4 Fig — (DOCX) [file pone.0272279.s004.docx]

**S5. Mean VIS over 48 hours for patients alive 48 hours after CICU admittance**
