# Supplementary material for: Vasoactive pharmacological management according to SCAI class in patients with acute myocardial infarction and cardiogenic shock
Source: PLoS One. 2022 Aug 4;17(8):e0272279. doi: 10.1371/journal.pone.0272279 (PMC9352108; doi:10.1371/journal.pone.0272279)
Supplement: S1 Table — (DOCX) [file pone.0272279.s006.docx]

**S4. Patients alive until day 3**

| **Alive** | **Day 0** | **Day 1** | **Day2** | **Day 3** |
| --- | --- | --- | --- | --- |
| **SCAI C** | 796 | 99.6 % (793) | 97% (771) | 94% (748) |
| **SCAI D** | 284 | 87% (248) | 76% (216) | 71% (203) |
| **SCAI E** | 169 | 82% (138) | 56% (94) | 44% (78) |
| **Total** | 1,249 | 94.3 % (1,179) | 87% (1,081) | 82% (1,029) |
