# Supplementary material for: Vasoactive pharmacological management according to SCAI class in patients with acute myocardial infarction and cardiogenic shock
Source: PLoS One. 2022 Aug 4;17(8):e0272279. doi: 10.1371/journal.pone.0272279 (PMC9352108; doi:10.1371/journal.pone.0272279)
Supplement: S2 Table — (DOCX) [file pone.0272279.s007.docx]

**S6. Patient characteristics based on the vasoactive strategy within each SCAI group**

| **SCAI class C** |  |  |  |  |  |
| --- | --- | --- | --- | --- | --- |
| **Variable** | **NE** | **DA** | **MIX NE/DA** | **MIX+AD** | **p-value** |
|  | N=197 | N=142 | N=367 | N=90 |  |
| Age | 65 (12) | 62 (11) | 65 (11) | 66 (11) | 0.046 |
| Gender | 155 (79%) | 116 (82%) | 307 (84%) | 70 (78%) | 0.39 |
| Hypertension | 104 (55%) | 57 (42%) | 176 (49%) | 42 (48%) | 0.17 |
| Ischemic heart disease | 58 (30%) | 31 (22%) | 85 (24%) | 31 (35%) | 0.054 |
| Myocardial infarction | 30 (16%) | 13 (9%) | 48 (13%) | 19 (22%) | 0.065 |
| Diabetes |  |  |  |  | 0.51 |
| no diabetes | 160 (84%) | 121 (88%) | 295 (83%) | 67 (77%) |  |
| diabetes type 1 | 4 (2%) | 1 (1%) | 8 (2%) | 2 (2%) |  |
| diabetes type 2 | 26 (14%) | 16 (12%) | 52 (15%) | 18 (21%) |  |
| Peripheral atherosclerotic disease | 13 (7%) | 6 (4%) | 24 (7%) | 6 (7%) | 0.77 |
| Chronic obstructive lung disease | 14 (7%) | 10 (7%) | 37 (10%) | 10 (11%) | 0.47 |
| stroke | 18 (9%) | 8 (6%) | 27 (8%) | 3 (3%) | 0.30 |
| Out of hospital cardiac arrest | 97 (49%) | 76 (54%) | 244 (66%) | 34 (38%) | <0.001 |
| Initial heart rate, min^-1^ | 84 (70-100) | 81 (70-93) | 80 (65-98) | 85 (72-110) | 0.11 |
| Initial systolic BP, mmHg | 85 (78-95) | 87 (80-94) | 87 (78-95) | 80 (70-90) | 0.005 |
| Initial lactate, mmol/L | 4.0 (2.5-6.8) | 4.0 (2.0-7.8) | 4.3 (2.6-8.3) | 6.0 (3.3-9.0) | 0.001 |
| Initial pH | 7.30  (7.24-7.36) | 7.31  (7.27-7.35) | 7.29  (7.25-7.34) | 7.25  (7.20-7.31) | <0.001 |
| Initial HCO3, mmol/L | 20.4  (18.7-22.1) | 20.7  (19-22.4) | 20.3  (18.5-22.1) | 18.6  (16.8-19.9) | <0.001 |
| Initial glucose, mmol/L | 10.5  (8.4-13.9) | 8.8  (7.3-10.8) | 9.1  (7.5-12.1) | 13.0  (10.4-16.5) | <0.001 |
| Revascularization | 183 (93%) | 135 (95%) | 328 (89%) | 80 (89%) | 0.14 |
| Culprit lesion |  |  |  |  | 0.036 |
| Left main | 18 (10%) | 9 (7%) | 17 (5%) | 10 (13%) |  |
| LAD | 96 (52%) | 63 (47%) | 142 (43%) | 36 (45%) |  |
| LCx | 30 (16%) | 29 (21%) | 63 (19%) | 9 (11%) |  |
| RCA | 39 (21%) | ´ 34 (25%) | 106 (32%) | 25 (31%) |  |
| Mechanical ventilation | 166 (85%) | 111 (78%) | 347 (95%) | 88 (98%) | <0.001 |
| Impella | 30 (15%) | 5 (4%) | 28 (8%) | 32 (36%) | <0.001 |
| VA-ECMO | 3 (2%) | 0 (0%) | 1 (0%) | 0 (0%) | 0.13 |
| IABP | 16 (8%) | 16 (11%) | 33 (9%) | 22 (24%) | <0.001 |

Data are presented as mean with standard deviation (SD), frequencies with percentages (%) or median with interquartile range of 25^th^ and 75^th^ (IQR).

| **SCAI class D** |  |  |  |  |  |
| --- | --- | --- | --- | --- | --- |
| **Variable** | **NE** | **DA** | **MIX NE/DA** | **MIX+AD** | **p-value** |
|  | N=63 | N=23 | N=83 | N=110 |  |
| Age | 72 (9) | 69 (11) | 68 (10) | 69 (11) | 0.14 |
| Gender | 42 (67%) | 16 (70%) | 63 (76%) | 79 (72%) | 0.67 |
| Hypertension | 31 (50%) | 13 (65%) | 47 (59%) | 59 (56%) | 0.61 |
| Ischemic heart disease | 18 (30%) | 11 (52%) | 22 (27%) | 37 (34%) | 0.16 |
| Myocardial infarction | 9 (15%) | 6 (29%) | 10 (12%) | 23 (21%) | 0.21 |
| Diabetes |  |  |  |  | 0.086 |
| no diabetes | 52 (85%) | 16 (80%) | 60 (76%) | 87 (84%) |  |
| diabetes type 1 | 2 (3%) | 2 (10%) | 2 (3%) | 0 (0%) |  |
| diabetes type 2 | 7 (11%) | 2 (10%) | 17 (22%) | 17 (16%) |  |
| Peripheral atherosclerotic disease | 5 (8%) | 2 (10%) | 9 (11%) | 13 (12%) | 0.88 |
| Chronic obstructive lung disease | 12 (20%) | 2 (11%) | 5 (6%) | 13 (12%) | 0.11 |
| stroke | 6 (10%) | 3 (14%) | 7 (9%) | 15 (14%) | 0.66 |
| Out of hospital cardiac arrest | 22 (35%) | 9 (39%) | 43 (52%) | 41 (37%) | 0.13 |
| Initial heart rate, min^-1^ | 90 (75-101) | 84 (65-120) | 89 (73-106) | 84 (73-100) | 0.87 |
| Initial systolic BP mmHg | 85 (70-95) | 88 (73-98) | 84 (77-95) | 81 (74-90) | 0.57 |
| Initial lactate, mmol/L | 4.5 (3.1-6.5) | 4.6 (2.4-7.0) | 4.9 (3.3-7.0) | 6.4 (4.9-9.4) | <0.001 |
| Initial pH | 7.29  (7.20-7.36) | 7.33  (7.27-7.38) | 7.25  (7.19-7.31) | 7.23  (7.18-7.28) | <0.001 |
| Initial HCO3, mmol/L | 18.7  (16.0-20.9) | 20.6  (19.1-22.5) | 19.2  (16.8-20.5) | 18.4  (16.1-20.0) | 0.074 |
| Initial glucose, mmol/L | 11.8  (9.6-14.3) | 12.1  (9.2-15.1) | 12.4  (10.0-15.1) | 13.0  (10.2-16.1) | 0.30 |
| Revascularization | 52 (83%) | 20 (87%) | 74 (89%) | 98 (89%) | 0.60 |
| Culprit lesion |  |  |  |  | 0.41 |
| Left main | 11 (21%) | 1 (5%) | 7 (9%) | 19 (19%) |  |
| LAD | 20 (38%) | 8 (40%) | 37 (50%) | 43 (44%) |  |
| LCx | 5 (10%) | 4 (20%) | 10 (14%) | 8 (8%) |  |
| RCA | 16 (31%) | 7 (35%) | 20 (27%) | 28 (29%) |  |
| Mechanical ventilation | 55 (87%) | 15 (65%) | 77 (93%) | 107 (97%) | <0.001 |
| Impella | 7 (11%) | 0 (0%) | 7 (8%) | 26 (24%) | 0.002 |
| VA-ECMO | 1 (2%) | 0 (0%) | 0 (0%) | 0 (0%) | 0.33 |
| IABP | 4 (6%) | 0 (0%) | 9 (11%) | 15 (14%) | 0.16 |

| **SCAI class E** |  |  |  |  |  |
| --- | --- | --- | --- | --- | --- |
| **Variable** | **NE** | **DA** | **MIX NE/DA** | **MIX+AD** | **p-value** |
|  | N=25 | N=2 | N=32 | N=107 |  |
| Age | 55 (13) | 68 (24) | 66 (11) | 65 (11) | <0.001 |
| Gender | 20 (80%) | 2 (100%) | 21 (66%) | 84 (79%) | 0.37 |
| Hypertension | 8 (33%) | 2 (100%) | 13 (43%) | 48 (49%) | 0.23 |
| Ischemic heart disease | 5 (21%) | 2 (100%) | 5 (17%) | 36 (35%) | 0.028 |
| Myocardial infarction | 3 (13%) | 1 (50%) | 3 (10%) | 15 (15%) | 0.45 |
| Diabetes |  |  |  |  | 0.20 |
| no diabetes | 16 (67%) | 0 (0%) | 23 (79%) | 74 (75%) |  |
| diabetes type 1 | 1 (4%) | 0 (0%) | 0 (0%) | 1 (1%) |  |
| diabetes type 2 | 7 (29%) | 2 (100%) | 6 (21%) | 24 (24%) |  |
| Peripheral atherosclerotic disease | 0 (0%) | 1 (50%) | 3 (10%) | 8 (8%) | 0.068 |
| Chronic obstructive lung disease | 1 (4%) | 0 (0%) | 3 (10%) | 11 (11%) | 0.76 |
| stroke | 1 (4%) | 0 (0%) | 0 (0%) | 2 (2%) | 0.73 |
| Out of hospital cardiac arrest | 12 (48%) | 1 (50%) | 16 (50%) | 40 (37%) | 0.53 |
| Initial heart rate, min^-1^ | 90 (80-109) |  | 80 (70-97) | 91 (80-108) | 0.19 |
| Initial systolic BP, mmHg | 80 (70-85) | 95 (80-110) | 80 (74-91) | 80 (69-90) | 0.52 |
| Initial lactate, mmol/L | 12.9 (9.8-15.0) | 14.3 (13.7-15.0) | 9.9 (7.9-13.2) | 12.0 (8.0-14.6) | 0.18 |
| Initial pH | 7.24  (7.07-7.32) | 7.14  (7.13-7.14) | 7.19  (7.11-7.28) | 7.16  (7.08-7.23) | 0.35 |
| Initial HCO3, mmol/L | 19.3  (14.6-22.0) | 16.6  (16.6-16.6) | 17.3  (15.6-20.0) | 15.9  (13.7-19.7) | 0.25 |
| Initial glucose, mmol/L | 15.6 (10.5-19.1) | 22.1 (15.7-28.5) | 12.9 (10.6-18.6) | 14.3 (9.4-19.8) | 0.64 |
| Revascularization | 21 (84%) | 1 (50%) | 29 (91%) | 100 (93%) | 0.095 |
| Culprit lesion |  |  |  |  | 0.91 |
| Left main | 4 (19%) | 0 (0%) | 7 (24%) | 23 (23%) |  |
| LAD | 10 (48%) | 0 (0%) | 12 (41%) | 39 (39%) |  |
| LCx | 1 (5%) | 0 (0%) | 3 (10%) | 6 (6%) |  |
| RCA | 6 (29%) | 1 (100%) | 7 (24%) | 32 (32%) |  |
| Mechanical ventilation | 23 (92%) | 2 (100%) | 31 (97%) | 106 (99%) | 0.22 |
| Impella | 6 (24%) | 0 (0%) | 12 (38%) | 32 (30%) | 0.54 |
| VA-ECMO | 12 (48%) | 0 (0%) | 8 (25%) | 25 (23%) | 0.069 |
| IABP | 2 (8%) | 0 (0%) | 2 (6%) | 21 (20%) | 0.17 |
