# Supplementary material for: Vasoactive pharmacological management according to SCAI class in patients with acute myocardial infarction and cardiogenic shock
Source: PLoS One. 2022 Aug 4;17(8):e0272279. doi: 10.1371/journal.pone.0272279 (PMC9352108; doi:10.1371/journal.pone.0272279)
Supplement: S3 Table — (DOCX) [file pone.0272279.s008.docx]

|  | Total | NE | DA | NE/DA | MIX+AD | p-value |
| --- | --- | --- | --- | --- | --- | --- |
| Death | **Total** | **NE** | **DA** | **NA/DA** | **MIX+AD** |  |
| SCAI C | **N=267** | **N=55** | **N=35** | **N=131** | **N=46** |  |
|  |  |  |  |  |  |  |
| Cause of death |  |  |  |  |  | 0.002 |
| Multi-organ Failure | 12% | 15% | 9% | 8% | 22% |  |
| Cardiac failure | 21% | 25% | 20% | 12% | 41% |  |
| Cerebral anoxia | 49% | 42% | 54% | 60% | 24% |  |
| Respiratory failure | 1% | 0% | 0% | 2% | 2% |  |
| Other | 9% | 11% | 6% | 8% | 9% |  |
| Missing | 8% | 7% | 11% | 10% | 2% |  |
|  |  |  |  |  |  |  |
| SCAI D | **N=165** | **N=40** | **N=10** | **N=49** | **N=66** |  |
|  |  |  |  |  |  |  |
| Cause of death |  |  |  |  |  | 0.002 |
| Multi-organ failure | 18% | 15% | 0% | 24% | 17% |  |
| Sepsis | 3% | 3% | 0% | 6% | 2% |  |
| Cardiac failure | 41% | 53% | 20% | 18% | 55% |  |
| Cerebral anoxia | 25% | 13% | 50% | 35% | 21% |  |
| Other | 9% | 13% | 30% | 8% | 5% |  |
| Missing | 4% | 5% | 0% | 8% | 2% |  |
|  |  |  |  |  |  |  |
| SCAI E | **N=136** | **N=18** | **N=1** | **N=27** | **N=90** |  |
|  |  |  |  |  |  |  |
| Cause of death |  |  |  |  |  | 0.39 |
| Multi-organ failure | 27% | 28% | 0% | 41% | 23% |  |
| Sepsis | 1% | 0% | 0% | 0% | 1% |  |
| Cardiac failure | 44% | 33% | 100% | 26% | 51% |  |
| Cerebral anoxia | 17% | 33% | 0% | 22% | 12% |  |
| Other | 10% | 6% | 0% | 7% | 11% |  |
| Missing | 1% | 0% | 0% | 4% | 1% |  |

**S8. Cause of death in each SCAI class divided according to vasoactive strategy**

8 patients receiving epinephrine only are excluded, they died within 24 hours.
