# Supplementary material for: Vasoactive pharmacological management according to SCAI class in patients with acute myocardial infarction and cardiogenic shock
Source: PLoS One. 2022 Aug 4;17(8):e0272279. doi: 10.1371/journal.pone.0272279 (PMC9352108; doi:10.1371/journal.pone.0272279)
Supplement: Attachment — Submitted filename: Vasoactive pharmacological management according to SCAI class in patients with.docx [file pone.0272279.s009.docx]

Vasoactive pharmacological management according to SCAI class in patients with

acute myocardial infarction and cardiogenic shock, despite being retrospective it is a good study. It includes important data, from a representative population through a detailed analysis of patients admitted to intensive care, from 2010 to 2017, in 2 centers that provide tertiary cardiac care for a population of 3.8 million people. Treatment with inopressors was evaluated in detail with regard to its indication in relation to the severity of the disease, its response, and the type of drug used. In my opinion, although only two centers were included, it is a quality work, providing important information regarding the treatment of critically ill patients with cardiogenic shock following acute myocardial infarction.
